# Integrating emergency medical services and palliative care: A nominal group technique

**DOI:** 10.4102/phcfm.v17i1.4891

**Published:** 2025-06-24

**Authors:** Caleb H. Gage, Liz Gwyther, Julia Ambler, Jan Burke, Katya Evans, Linley Holmes, René Krause, Kaleb Lachenicht, Danielle Lincoln, Kerene Payne, Mpho Ratshikana-Moloko, Charnelle Stander, Willem Stassen

**Affiliations:** 1 Division of Emergency Medicine, Faculty of Health Sciences, University of Cape Town, Cape Town, South Africa; 2 Division of Interdisciplinary Palliative Care and Medicine, Faculty of Health Sciences, University of Cape Town, Cape Town, South Africa; 3 Umduduzi-Hospice Care for Children, Durban, South Africa; 4 Department of Paediatrics, Faculty of Health Sciences, University of KwaZulu-Natal, Durban, South Africa; 5 Department of Family Medicine, Faculty of Health Sciences, University of KwaZulu-Natal, Durban, South Africa; 6 Netcare 911, Johannesburg, South Africa; 7 Department of Health, Mitchells Plain District Hospital, Cape Town, South Africa; 8 Department of Health, Groote Schuur Hospital, Cape Town, South Africa; 9 Rocket Helicopter Emergency Medical Service, Johannesburg, South Africa; 10 Hospice, University of the Witwatersrand, Johannesburg, South Africa; 11 Chariot Health, Cape Town, South Africa; 12 Department of Health, Chris Hani Baragwanath Academic Hospital, Johannesburg, South Africa; 13 Centre for Palliative Care, Faculty of Health Sciences, University of the Witwatersrand, Johannesburg, South Africa; 14 Soweto Comprehensive Cancer Centre, Johannesburg, South Africa

**Keywords:** paramedic, ambulance, EMS, palliative care, end-of-life, person-centred care, healthcare system integration, South Africa

## Abstract

**Background:**

The need for integrated healthcare has been increasingly recognised because of mounting challenges associated with the proliferation of injuries and noncommunicable diseases. A developing example of integration is between Emergency Medical Services (EMS) and palliative care. Despite recommendations for integration in South Africa (SA), these services remain segregated.

**Aim:**

This study aimed to develop and prioritise approaches facilitating EMS and palliative care system integration within SA.

**Setting:**

An online meeting was held with SA EMS and palliative care experts.

**Methods:**

A nominal group technique was employed to answer the question, ‘What do you think should be done to most effectively integrate EMS and palliative care services in SA?’ Answers were categorised, awarded scores by participants, and ranked according to impact and feasibility.

**Results:**

The following categories were generated: Awareness, Education, Community Engagement, Communication and Information Sharing, Stakeholder Collaborations, Alternative Pathways and Approaches, Research, Funding, Policy Development and Governance. The top five individual approaches were: (1) enable EMS to administer already prescribed medications, (2) Emergency Medical Services undergraduate training in palliative care, (3) improve EMS recognition of signs of dying at the end-of-life, (4) palliative care awareness for the EMS community, and (5) palliative care awareness for in-hospital healthcare providers, particularly those in emergency medicine.

**Conclusion:**

The categories developed in this study should be used to guide EMS and palliative care integration in SA. Future research should aim at establishing the safety and efficacy of these interventions.

**Contribution:**

This study provides a structured approach to integrating EMS and palliative care in SA, enhancing holistic care for patients with palliative needs.

## Introduction

The need for integrated healthcare has been increasingly recognised as health systems face mounting challenges from ageing populations, unhealthy lifestyles, urbanisation and subsequent proliferation of injuries and noncommunicable diseases (NCDs).^1^ Fragmented modern healthcare exacerbates these challenges through inefficient resource use leading to inflated costs, poor quality of care and decreased patient satisfaction.^1^ These challenges are particularly concerning in low-to-middle income countries (LMICs), which contain disproportionately high disease burdens with concurrent resource limitations.^2^ The World Health Organization (WHO) global strategy on Integrated People-Centred Health Services (IPCHS) states, ‘The focus on hospital-based and self-contained “silo”: curative care models undermines the ability of health systems to provide universal, equitable, high-quality and financially sustainable care’.^1^ Integrated health systems, defined as ‘the coordination of health services and the collaboration amongst provider organisations to establish an effective health system’,^3^ have been recommended to improve accessibility, affordability and quality of care, particularly for those with complex healthcare needs.^4^

A developing example of healthcare integration is collaboration between Emergency Medical Services (EMS) and palliative care.^5^ Palliative care itself is integrative in nature, as it applies a multidisciplinary approach to those with complex, life-limiting illnesses.^6^ The WHO has specifically recommended palliative care integration across multiple systems as part of its global response to NCDs to meet the increasing global demand for palliative care.^7^ Several studies have demonstrated the effectiveness of EMS and palliative care integration in this regard as it enhances patient satisfaction, improves quality of life, avoids unnecessary hospital admissions and associated costs and improves access to palliative care.^8,9,10,11^ In LMICs such as South Africa (SA), where healthcare resources are constrained, integrating EMS with palliative care could improve accessibility while optimising limited resources.

In SA, these benefits have been noted by EMS^12^ and palliative care providers^13^ as well as patients and family members.^14^ These stakeholder groups have recommended EMS and palliative care integration.^12,13,14^ Despite these findings, EMS and palliative care systems remain ‘siloed’ in SA, and no clear guidance exists for integration. Thus, there is a need to develop implementable approaches facilitating integration. Given the resource constraints in SA, there is a further need to prioritise approaches with the greatest potential impact and feasibility. Therefore, the aim of this study was to develop and prioritise approaches facilitating EMS and palliative care integration within SA.

## Research methods and design

### Design

A nominal group technique (NGT), involving EMS and palliative care experts, was employed. A complex adaptive system (CAS) theory of healthcare underpins the study.^15,16^ The CAS theory views healthcare as an open, self-organising system consisting of multiple agents with complex interactions analogous to a living organism, which constantly adapts to change.^15,16^ Thus, rather than seeking permanent solutions to the problem of segregated EMS and palliative care, we sought to identify facilitators of integration, which may be applied cross-contextually in SA.

### Setting

The SA health system, including EMS, is divided into private and state sectors. The state sector, operated by the government, provides healthcare to all citizens. The private sector is restricted to those with medical insurance or other financial means. Emergency Medical Services care is provided using a paramedic-led rather than a physician-led approach.^12^ Formal higher education (HE) training is required to register as an EMS provider; however, as this was a recent requirement, many providers with vocational training remain registered and practicing.^17^ Higher education training ranges from 1 (assistant) to 4 years (practitioner) in duration. Palliative care in SA is frequently delivered *via* nongovernmental organisations, although there has been significant growth in state sector palliative care provision.^18,19^ Although palliative care integration has improved in many medical disciplines, equitable access to palliative care remains a significant challenge.^20^

### Study population and sampling strategy

Emergency Medical Services and palliative care experts meeting inclusion criteria were purposefully sampled. ‘Palliative care experts’ were qualified medical nurses and doctors with a minimum of 5 years postgraduate experience and a postgraduate HE qualification in a palliative care-related field (i.e. Postgraduate Diploma in Palliative Care). ‘EMS experts’ were advanced life support (ALS) providers with HE qualifications (National Diploma, Bachelor of Technology, Bachelor of Health Sciences in Emergency Medical Care), a minimum of 5 years postgraduate EMS experience and a postgraduate HE qualification in an EMS or palliative care-related field (i.e. Postgraduate Diploma in Palliative Care or Emergency Medicine). The EMS and palliative care experts without experience and qualifications in SA were not included. A minimum postgraduate experience of 5 years was selected to ensure adequate experience and expert-level insights. Participants were individually invited to the study *via* email. A brief study description and a proposed meeting time were presented and willing participants signed a consent form.

### Data collection

One virtual nominal group meeting was performed using Microsoft Teams (Microsoft Corporation, United States) with audio-recording and transcription functions enabled. A single meeting was held for logistical feasibility and as limited information is available to guide data analysis when multiple meetings are used in a single study.^21^ A virtual platform was used as participants were broadly distributed across SA. The CG and WS facilitated a pilot meeting with LG as the palliative care expert and an additional EMS expert to ensure procedural quality. After this pilot, the procedural schedule (Online Appendix 1) was finalised. Unique findings from the pilot were presented in the formal meeting for discussion and were ultimately included in the final list of approaches.

Prior to the meeting, prereading was sent to participants *via* email introducing the study topic, aim, question and an explanation of the study process. The CG facilitated the meeting with WS and LG assisting. As described by McMillan et al.,^21^ the study was conducted in four consecutive phases. Time allocations for each phase were flexible based on participants’ needs and discussions:

- Silent Idea Generation: As prereading had been sent, participants were given 5 min to silently and individually record as many answers as possible to the question, ‘what do you think should be done to most effectively integrate EMS and palliative care services in SA?’ During this time, the research team remained silent.
- Round Robin Discussion: Participants individually presented a single answer at a time in a round robin fashion until all ideas were exhausted. Answers were not discussed but simply recorded by CG for all participants to see. Answers were placed into predefined categories (Online Appendix 2) based on previous SA research,^12,13,14^ the pilot meeting and health system integration literature.^22^ This phase was allocated 30 min.
- Clarification: In this phase, 1 h was allocated for discussion concerning each answer. Participants were given the opportunity to ask questions about the submitted answers and clarify their meaning. Based on the discussions, similar answers were combined, some were excluded, and new answers were recorded. After each answer was discussed, a final list of answers was produced.
- Ranking: Participants were asked to individually rank each answer according to its impact and feasibility in the SA setting. This was done *via* email with a link to a Microsoft Forms (Microsoft Corporation, United States) survey (Online Appendix 3). Impact and feasibility were divided into low, moderate and high, which were awarded scores of 1, 2 and 3, respectively. Thus, for example, an idea could be ranked as having high impact (3) but low feasibility (1). Scores were then totalled across all participants, and ideas arranged from highest to lowest score, producing a prioritised list. Average impact and feasibility scores were also calculated to place ideas in final categories of low, moderate or high. For example, if an idea averaged a score of 2.6 for impact, this was rounded to 3, and the idea was placed in the high-impact category. For the purposes of this study, impact referred to the effectiveness of an approach in integrating EMS and palliative care, and feasibility referred to ease of implementation in the SA context. Thus, a high-impact approach would be very effective and a highly feasible approach would be easily implementable.

### Data analysis

Data analysis was performed according to the recommendations of McMillan et al.^21^:

- Descriptive Analysis of Raw Data: Answers and their priorities were descriptively analysed in terms of participant scoring.
- Categorical Analysis of Raw Data: The answers generated by participants were coded into categories by CG. Researcher triangulation was performed between the authorship where codes and categories were discussed, refined and finalised. Member checking of these categories was performed to ensure the intended meaning of the participants was retained.
- Analysis of Secondary Coded Data: Categories were ranked by averaging the total scores of answers within each category.
- Qualitative Analysis: The transcript of the meeting was deductively analysed according to the developed categories to provide further context, such as reasons for answers.

Summary descriptive statistics (means, standard deviations) were used to calculate individual answer and category scores using SPSS Statistics for Windows version 28.0 (IBM Corp., United states).

### Ethical considerations

Ethical clearance to conduct this study was obtained from the University of Cape Town’s Human Research Ethics Committee (HREC) (No. 453/2024).

## Results

**FIGURE 1:**
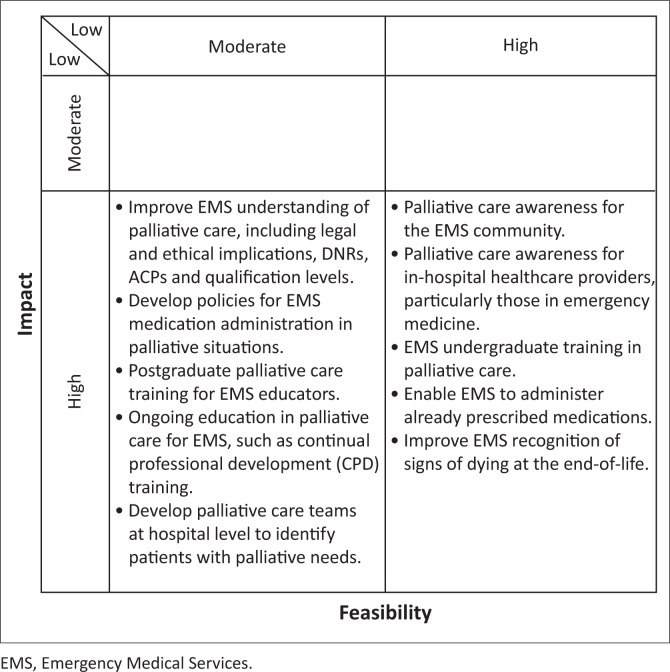
Prioritisation matrix.

Nine experts participated in the meeting (Table 1), which lasted 2 h 27 min. Seven participants knew CG from the previous research participation in EMS^12^ and palliative care expert interviews,^13^ and all were aware that the study formed part of his postgraduate studies. No participants dropped out of the study or refused participation. All participants completed each phase of the procedure. In total, 52 answers were generated and ranked (Table 2). Answers fell into 10 categories, as demonstrated in Table 3. Based on average impact and feasibility scores, ideas were plotted on a prioritisation matrix (Figure 1).

**TABLE 1:** Expert panel member and research team details.

Participants and researchers	Area of expertise	Years of experience	Qualifications	SA province
**Participant #1**	Palliative Care	10	BCur, PG Dip Palliative Medicine, MPhil Palliative Medicine	Western Cape
**Participant #2**	Palliative Care	25	MBChB, MRCGP, PG Dip Palliative Medicine, DCH	KwaZulu-Natal
**Participant #3**	Palliative Care	20	MBChB, MMed Family Medicine, MPhil Palliative Medicine, PhD Palliative Medicine	Western Cape
**Participant #4**	EMS	10	BTEMC, MPhil Emergency Medicine	Gauteng
**Participant #5**	EMS	13	BTEMC, PG Dip Health Science Education, PG Dip Medical Simulation, MSc Health Science Education	Gauteng
**Participant #6**	EMS	11	BTEMC, MPhil Emergency Medicine	Gauteng
**Participant #7**	Palliative Care	14	MBChB, DTM&H, MPhil Palliative Medicine	Gauteng
**Participant #8**	Palliative Care	35	MBChB, MPhil Palliative Medicine	Gauteng
**Participant #9**	Palliative Care and Emergency Medicine	16	MBChB, MMed Emergency Medicine, PG Dip Palliative Medicine	Western Cape
**Pilot study participant**	EMS and Palliative Care	14	BTEMC, PG Dip Higher Adult Education, PG Dip Palliative Medicine, MPhil Palliative Medicine	Western Cape
**WS**	EMS	14	BTEMC, PG Dip Applied Ethics, MPhil Emergency Medicine, PhD Emergency Medicine	Western Cape
**LG**	Palliative Care	31	MBChB, MMed Family Medicine, PG Dip Palliative Medicine, MSc Palliative Medicine, PhD Palliative Medicine	Western Cape
**CG**	EMS	11	BTEMC, MPhil Emergency Medicine, Palliative Care Short Course*	Gauteng

BCur, Bachelor of Nursing Science; PG Dip, Post-Graduate Diploma; MPhil, Master of Philosophy; MBChB, Bachelor of Medicine and Bachelor of Surgery; MRCGP, Membership of the Royal College of General Practitioners; DCH, Diploma in Child Health; MMed, Master of Medicine; PhD, Doctor of Philosophy; BTEMC, Bachelor of Technology in Emergency Medical Care; MSc, Master of Science; DTM&H, Diploma in Tropical Medicine and Hygiene; SA, South Africa; EMS, Emergency Medical Services.

* , 10-week course introducing basic palliative care principles and management.

**TABLE 2:** Generated answers in rank order.

Ideas	Category	Total impact score	Mean	s.d.	Total feasibility score	Mean	s.d.	Total score
Palliative care awareness for the EMS community.	Awareness	26	2.80	0.33	24	2.67	0.71	50
Palliative care awareness for in-hospital healthcare providers, particularly those in emergency medicine.	Awareness	26	2.89	0.33	23	2.56	0.53	49
EMS undergraduate training in palliative care.	Education	24	2.67	0.50	25	2.78	0.44	49
Enable EMS to administer already prescribed medications.	Alternative pathways and approaches	24	2.67	0.71	24	2.67	0.50	48
Improve EMS recognition of signs of dying at the end-of-life.	Education	24	2.67	0.50	23	2.56	0.73	47
Improve EMS understanding of palliative care – including legal and ethical implications, DNRs, ACPs, qualification levels.	Education	25	2.78	0.44	22	2.44	0.88	47
Develop policies for EMS medication administration in palliative situations.	Policy development	25	2.78	0.44	21	2.33	0.50	46
Postgraduate palliative care training for EMS educators.	Education	25	2.78	0.44	21	2.33	0.87	46
Ongoing education in palliative care for EMS such as continual professional development (CPD) training.	Education	24	2.67	0.71	22	2.44	0.73	46
Develop palliative care teams at hospital level to identify patients with palliative needs.	Alternative pathways and approaches	26	2.89	0.33	20	2.22	0.97	46
Develop EMS palliative care networking teams.	Stakeholder collaborations	24	2.67	0.50	21	2.33	0.87	45
Provide palliative care advice for EMS at the point of care.	Communication and information sharing	24	2.67	0.50	21	2.33	0.71	45
EMS training in subcutaneous medication administration.	Education	22	2.44	0.73	23	2.56	0.73	45
Train EMS in patient and family member communication.	Education	23	2.56	0.53	22	2.44	0.53	45
EMS palliative care curriculum development.	Education	24	2.67	0.50	21	2.33	0.87	45
Develop guidelines and standard operating procedures for EMS at the point of care – including who to contact for advice.	Policy development	25	2.78	0.44	20	2.22	0.83	45
Provide support for EMS to fulfil advance care plans by including information (instructions, contact details) specific to EMS in the plans.	Communication and information sharing	24	2.67	0.71	21	2.33	0.71	45
Collaboration between EMS and palliative care societies.	Stakeholder collaborations	22	2.44	0.73	23	2.56	0.53	45
24/7 availability of palliative care contacts for patients and family members.	Community engagement	26	2.89	0.33	19	2.11	0.60	45
Develop a model for EMS and palliative care integration at various healthcare system levels and within various EMS environments.	Research	26	2.89	0.33	18	2.00	0.71	44
Develop mechanisms for EMS to handover patient care in palliative situations to family members.	Alternative pathways and approaches	23	2.56	0.53	21	2.33	0.87	44
Develop EMS support tools to assist in the identification of patients with previously unrecognised palliative needs.	Alternative pathways and approaches	24	2.67	0.71	20	2.22	0.83	44
Adjust private sector EMS funding models relating to medical insurance.	Funding	24	2.67	0.50	19	2.11	0.60	43
Define processes for EMS linking palliative situations with outpatient services, community healthcare and community oriented primary care.	Alternative pathways and approaches	25	2.78	0.44	18	2.00	0.87	43
Develop palliative care committees inclusive of EMS, hospice and in-hospital management at all levels of care.	Stakeholder collaborations	23	2.56	0.53	20	2.22	0.83	43
Postgraduate palliative care training for EMS providers.	Education	23	2.56	0.53	20	2.22	0.83	43
Enable early EMS recognition of palliative cases.	Education	24	2.67	0.71	19	2.11	0.78	43
Engage community concerning specific palliative care needs.	Community engagement	21	2.33	0.71	21	2.33	0.50	42
Establish EMS palliative care case reviews and debriefing sessions for quality improvement.	Governance	24	2.67	0.50	18	2.00	0.71	42
Include EMS in interdisciplinary palliative care education.	Education	22	2.44	0.73	19	2.11	0.60	41
Establish cross-disciplinary communication and information-sharing with EMS, palliative care, in-hospital providers.	Communication and information sharing	23	2.56	0.53	18	2.00	0.50	41
EMS and palliative care networking with locally available resources.	Stakeholder collaborations	23	2.56	0.53	18	2.00	0.50	41
Allocate budget for palliative care within EMS – education, policy and guideline development, service provision.	Funding	25	2.78	0.44	15	1.67	0.50	40
Perform ongoing EMS and palliative care research concerning efficacy of integration.	Research	20	2.22	0.83	20	2.22	0.67	40
Provide palliative care awareness and education for the public.	Community engagement	22	2.40	0.73	18	2.00	1.00	40
Develop care packages for EMS in palliative situations based upon scope of practice.	Alternative pathways and approaches	21	2.33	0.71	19	2.11	0.78	40
Include EMS within advance care plans.	Communication and information sharing	24	2.67	0.71	16	1.78	0.83	40
Include EMS in palliative care policies to link services and prioritise palliative care cases.	Policy development	21	2.33	0.71	18	2.00	0.71	39
Perform EMS and palliative care integration cost-benefit analyses.	Research	20	2.22	0.67	18	2.00	0.71	38
Define alternative care pathways for EMS in palliative situations.	Alternative pathways and approaches	22	2.44	0.53	16	1.78	0.83	38
Make advance care plans available for EMS at the point of care.	Communication and information sharing	23	2.56	0.73	15	1.67	0.71	38
Allocate finances for EMS in palliative care in the public sector.	Funding	24	2.67	0.50	13	1.40	0.58	37
Create specialist out-of-hospital palliative care teams inclusive of EMS and/or non-EMS staff.	Alternative pathways and approaches	21	2.33	0.87	16	1.78	0.83	37
Develop an EMS community care approach.	Alternative pathways and approaches	20	2.22	0.67	17	1.89	0.78	37
Have EMS providers spend time in palliative care practices.	Stakeholder collaborations	21	2.33	0.87	16	1.78	0.67	37
Recognise palliative care trained EMS providers.	Governance	21	2.33	0.71	15	1.67	0.71	36
Provide legal and ethical support for EMS in palliative situations.	Governance	20	2.22	0.83	16	1.78	0.97	36
Improve EMS scope of practice for EMS at the Health Professions Council of South Africa (HPCSA).	Governance	23	2.56	0.53	12	1.33	0.50	35
EMS and palliative care decision-maker collaboration at a national level.	Stakeholder collaborations	22	2.44	0.73	12	1.33	0.50	34
Develop telehealth systems for patients, family members and EMS.	Alternative pathways and approaches	18	2.00	0.50	13	1.44	0.73	31
Register advanced EMS providers in palliative care at HPCSA.	Governance	20	2.22	0.67	11	1.22	0.44	31
Develop policies for EMS to independently prescribe medications.	Policy development	16	1.78	0.83	13	1.44	0.73	29

Note: Answers are ranked from the highest to lowest priority based on total score, which is the sum of total impact and feasibility scores. As there were nine participants with the highest rating score of 3 (high) for each answer, the highest possible impact and feasibility scores were 27, while the highest possible total score for an idea was, therefore, 54. Mean impact and feasibility scores refer to the average of individual scores awarded by participants.

s.d., standard deviation; EMS, Emergency Medical Services.

**TABLE 3:** Categories in rank order.

Categories	Number of ideas	Mean total impact	s.d.	Mean total feasibility score	s.d.	Mean total score	s.d.
Awareness	2	26.00	0.00	23.50	0.71	49.50	0.71
Education	11	23.64	1.03	21.55	1.81	45.18	2.23
Community engagement	3	23.00	2.65	19.33	1.53	42.33	2.52
Communication and information sharing	5	23.60	0.55	18.20	2.77	41.80	3.11
Stakeholder collaborations	6	22.50	1.05	18.33	3.93	40.83	4.49
Alternative pathways and approaches	10	22.40	2.46	18.40	3.10	40.80	5.14
Research	3	22.00	3.46	18.67	1.15	40.67	3.06
Funding	3	24.33	0.58	15.67	3.06	40.00	3.00
Policy development	4	21.75	4.27	18.00	3.56	39.75	7.80
Governance	5	21.60	1.82	14.40	2.88	36.00	3.94

Note: Categories are ranked from highest to lowest priority based on mean total score.

s.d., standard deviation.

This figure includes the top 10 ranked ideas. A complete figure may be found in Online Appendix 4 Table A4-1.

### Awareness

Participants suggested palliative care awareness for both EMS and in-hospital healthcare providers. These approaches were awarded the highest impact scores and perceived as highly feasible. Thus, the awareness category was ranked as the highest overall priority in this study, as participants observed the lack of palliative care knowledge even among medical providers:

‘We need to define palliative care a bit better in the prehospital setting … If we [*EMS*] think palliative care, we just think about the stage four cancer getting morphine. That’s essentially EMS’s knowledge of palliative care.’ (Participant #6, EMS, Gauteng)

### Education

Ranked second overall, educational approaches to integration formed the largest category of discussion. Participants recommended palliative care training for EMS at all levels (undergraduate, postgraduate and ongoing professional development) and emphasised the need for curriculum development. As part of this curriculum, specific items, such as subcutaneous medication administration, were highlighted. The highest ranked educational approaches, prioritised as high impact, high feasibility, were EMS undergraduate training in palliative care and to improve EMS recognition of signs of dying at the end-of-life:

‘The training and the education of the [*EMS*] providers is so important … at an undergraduate level. I think it must start there.’ (Participant #8, Palliative Care, Gauteng)

‘So [*EMS*] education around recognising the signs of dying, and … using the subcutaneous route instead of intravenous access.’ (Participant #1, Palliative Care, Western Cape)

### Community engagement

Engaging with local communities was recommended by participants to identify specific palliative care needs. Palliative care awareness and education within local communities was also recommended, as participants found that many communities often misunderstood palliative care. Overall, the ideas within this category were ranked as having moderate impact and moderate feasibility. The highest ranked approach was 24/7 availability of palliative care contacts for patients and family members:

‘[*W*]ith the awareness and community, I think a lot of patients and family sees it [*palliative care*] as you giving up. Let them understand it’s not you’re giving up … it’s just a different type of care …’ (Participant #4, EMS, Gauteng)

‘So they must have a way for the patient to communicate with [*the palliative care team*] … if they don’t, they are going to phone EMS because there’s no one else to phone … in hours and then after hours.’ (Participant #9, Palliative Care and Emergency Medicine, Western Cape)

### Communication and information sharing

Several ideas developed by participants involved enhancing communication and information sharing between EMS and palliative care systems. Significant emphasis was placed on the inclusion of EMS within advance care planning as a support tool for EMS providers at the point of care. All approaches within this category were ranked as high impact, moderate feasibility. The highest ranked approaches were to provide palliative care advice for EMS at the point of care and to provide support for EMS to fulfil advance care plans by including information specific to EMS in the plans:

‘It’s developing the culture of conversation within the EMS spaces and … not just within the EMS space … because palliative care is gonna happen across multiple different spaces and that would require loads of communication …’ (Participant #5, EMS, Gauteng)

‘The advanced care planning documents and recognition of those documents when they do exist is really important … We’ve had quite a few cases where there are documents in place … and then when they [*EMS*] arrive … the patient still ends up going to hospital.’ (Participant #1, Palliative Care, Western Cape)

### Stakeholder collaborations

Various collaborations between EMS and palliative care stakeholders were recommended to promote integration. Stakeholders included EMS, palliative care, hospice and in-hospital healthcare providers, managers, national decision-makers and societies. These collaborations were recommended at all levels, from individual providers to national stakeholders. The top ranked approaches within this category were collaboration between EMS and palliative care societies and to develop EMS palliative care networking teams. Each of these fell into the high impact, moderate feasibility section of the prioritisation matrix:

‘So what I would like to see is perhaps at a district level the development of palliative care committees … Representatives from district hospitals, from the EMS, from pharmacy, from hospice services from … the hospital management.’ (Participant #2, Palliative Care, KwaZulu Natal)

‘[*S*]o when you talk about stakeholder engagement … we need all levels of care to be involved here.’ (Participant #7, Palliative Care, Gauteng)

### Alternative pathways and approaches

This category was the second largest and referred to differing approaches to patient care within EMS and palliative care systems than the current norm. For example, one approach was to develop mechanisms for EMS hand over of patient care in palliative situations to family members. Currently, EMS providers are obligated to handover care to a medical provider and no appropriate mechanism exists for treatment without conveyance.^13^ The highest ranked approach in this category, which was ranked as high impact, high feasibility, was to enable EMS to administer already prescribed medications:

‘I like to call it, not an on-scene discharge … but handover of care to family and the home-based care team who might not be on premises, but you are still handing over care to them.’ (Participant #9, Palliative Care and Emergency Medicine, Western Cape)

### Research

The need for ongoing research was emphasised by participants as important to monitor the efficacy of integration. The need for cost-benefit analyses was specifically highlighted. The top ranked approach within this category was to develop a model for EMS and palliative care integration at various healthcare system levels and within various EMS environments. This was ranked as high impact, moderate feasibility:

‘I think [*research is*] important for us as work starts with the integration … to be able to start documenting lessons that we are learning because we are going to have to probably have a model that might work in [*some*] areas and not work in others, so there’s going to be a lot of contextualisation.’ (Participant #7, Palliative Care, Gauteng)

### Funding

Participants recommended adjusting current EMS funding models and allocating funds for education, policy development and service provision in both state and private sectors to assist integrative efforts. The top ranked approach in this category was to adjust private sector EMS funding models relating to medical insurance. Currently, in SA, medical insurance companies do not reimburse EMS in instances of patient nonconveyance. While each idea in this category ranked highly in terms of impact, feasibility scores were relatively low, particularly for the allocation of funds in the state sector:

‘Let’s be very specific about where the budget allocations need to be. It needs to be for education, for service provision, for policy development.’ (Participant #3, Palliative Care, Western Cape)

### Policy development

The need for policies, guidelines and standard operating procedures for EMS providers in palliative situations was discussed by participants with a focus on medication administration and EMS linking patients with other services (i.e. hospice). It was also noted that existing palliative care policies do not mention EMS. The highest ranked approach for policy development was to develop policies for EMS medication administration in palliative situations. While this category was ranked low overall, this was primarily because of the low overall score of developing policies for EMS to independently prescribe medications:

‘[*G*]uidelines would be very key … we’ve got a lot of policies, I don’t even think our palliative care policies have actually recognised the need for us to … link together with EMS.’ (Participant #7, Palliative Care, Gauteng)

### Governance

This category largely consisted of macrolevel approaches involving specialist EMS provider registration with the Health Professions Council of South Africa (HPCSA), legal frameworks and increasing EMS scope of practice for palliative situations. Because of moderate and low feasibility scores of ideas within this category, governance was ranked last overall. The highest ranked approach within this category with a high impact, moderate feasibility score, was to establish EMS palliative care case reviews and debriefing sessions for quality improvement:

‘[*T*]hose EMS providers that have had advanced training in palliative care need to be recognised by the boards or their councils.’ (Participant #3, Palliative Care, Western Cape)

## Discussion

Healthcare integration literature has identified several components within integration frameworks: Funding, Colocation, Communication, Community Engagement, Context, Culture, Governance, Defining Roles and Shared Goals, Stakeholder Management, Technological Connectivity, Awareness and Education.^22^ Although some differing categories were developed in our study, participant answers aligned well overall. Categories not explicitly developed in our study were Colocation, Culture, Defining Roles and Shared Goals and Technological Connectivity. However, elements of these were implied within specific approaches. For example, establishing cross-disciplinary communication and information-sharing would require technological connectivity and colocation to varying degrees. Furthermore, educational interventions have been previously recommended to impact EMS and palliative care cultures.^14,23^ Additional categories noted in our study (Research, Policy Development, Alternative Pathways and Approaches) likewise contain similar elements as they would involve defining roles and shared goals. Thus, given their alignment with international healthcare integration frameworks and specificity to the SA setting, the categories developed here represent an appropriate foundation for EMS and palliative care integration in SA.

The approaches developed in our study may be achieved at macro- (national systems and policies), meso- (regional, district and organisational) and micro- (individual provider) levels (see Online Appendix 5 Table A5-1).^4,24^ This was demonstrated in a similar study aimed at implementing ‘palliative paramedicine’ in Australia.^24^ Within this Delphi study, 32 components for EMS and palliative care integration, divided into macro-, meso- and microlevel reached consensus.^24^ Although several specific interventions differ between this Delphi study and the present NGT, all categories of approaches are aligned.^24^

Considering the resource-limited LMIC context of SA, there is a need to focus on feasible, high impact and low-cost approaches to integration. Experts in this study identified several such approaches, which we highly recommend for immediate implementation: (1) enable EMS to administer already prescribed medications, (2) EMS undergraduate training in palliative care, (3) improve EMS recognition of signs of dying at the end-of-life, (4) palliative care awareness for the EMS community, and (5) palliative care awareness for in-hospital healthcare providers, particularly those in emergency medicine.

Currently, EMS providers do not typically administer medications that have already been prescribed, particularly if these medications fall outside of their scope of practice.^25^ An example provided by one participant was that of a patient with oral morphine at home who has not self-administered because of a misunderstanding of when the medication should be used. Emergency Medical Services are then contacted because of the patient’s distress – a common reason for EMS and palliative situation intersection.^26,27^ In this case, EMS should be empowered to assist with the self-administration of the oral morphine without subsequent conveyance to a hospital should the symptoms be relieved. This approach may allow many patients to be safely treated in the setting of their choice, which is frequently home.^28,29^ Unnecessary costs to both the patient and healthcare system may be prevented through avoidance of inappropriate conveyance.^30^

Educational approaches facilitating EMS and palliative care integration are the most frequently emphasised in the literature^5,31,32,33^ and were the most frequently highlighted approaches in our study. Emergency Medical Services undergraduate training in palliative care has been recommended internationally^23^ and in SA.^13^ In Australia, Juhrmann et al.^23^ recommended this as an approach to facilitate cultural change within EMS from a curative approach to one which recognises patients with supportive needs in different contexts. A study interviewing SA patients and family members in palliative situations argued this cultural change to a person-centred approach would be beneficial, not only in palliative situations but also emergency scenarios.^14^ However, cultural change will not arise from simply receiving palliative care training. Rather, palliative care principles should be embedded in the approach to care for all patients. For example, incorporating the palliative principle of holistic care to the EMS mindset would be beneficial in both palliative and emergency situations. We concur with Juhrmann et al.^23^ that undergraduate EMS training in palliative care should not detract from the EMS emergency response function, but rather enhance it. Such training should not be aimed at producing ‘paramedic palliative care providers’, but rather producing a cultural shift to person-centred care, which would enhance both emergency and palliative care provision.

Spreading awareness of palliative care among EMS and other healthcare providers, represents a simple, low-cost approach to service integration. One area in which EMS require assistance is identifying palliative situations.^34,35^ Because of their training, EMS providers tend to view all situations from an emergency perspective and are often not aware of alternative approaches.^12^ Thus, by spreading awareness of an alternative (palliative) approach, EMS recognition of palliative situations may be improved. This may be achieved through CPD training, dissemination of EMS and palliative care research, inclusion of EMS in palliative care conferences, meetings, strategic planning sessions and *vice versa*. We recommend each of these opportunities for awareness be explored and further innovations be developed.

While we recommend these highly impactful and feasible approaches, it should be recognised that even within SA, varying contexts exists between regions as some areas contain greater EMS and palliative care resources than others.^36,37,38^ Thus, the feasibility of these approaches will likely vary dependent on area. This may explain much of the variance in feasibility scores awarded by participants as they function in diverse contexts (see approaches with s.d. > 0.75). As a result, there is a need for flexibility in the application of these approaches. From our findings, we suggest there is no ‘one-size-fits-all’ approach to EMS and palliative care integration in SA, but rather unique priorities may develop in various contexts. This is potentially counterintuitive to the current EMS system, which tends to apply a ‘one-size-fits-all’ approach, applying curative care principles regardless of situation type.^14^ However, while unique priorities may develop, the categories of approaches presented here remain a reasonable starting point. Interestingly, no approaches were ranked as having low impact. Thus, each approach may be beneficial and should be considered cross-contextually.

While discussing the integration of EMS and palliative care, participants cautioned against palliative care services simply ‘passing on their work’ to EMS. South Africa EMS providers have likewise cautioned against performing palliative care in isolation.^12^ While integration between the services is necessary, the need to establish clear roles and boundaries is an important consideration^39^ and has been highlighted in healthcare integration literature.^40,41^ Future research is necessary to identify these roles and boundaries in various SA settings. Along with SA EMS and palliative care providers,^12,13^ we suggest the EMS role in palliative situations is to identify palliative needs, provide initial containment and link patients with palliative care providers.^42^ Additionally, EMS and palliative care integration should not be viewed as a *panacea*, but rather as part of an approach to improve palliative care provision in the country.^39^ Thus, ongoing research into EMS and palliative care integration must be performed to monitor its safety, efficacy and unintended consequences.

Our findings align with the WHO IPCHS strategy which recommends empowering and engaging people, strengthening governance and accountability, reorienting the model of care and coordinating services.^1^ These recommendations take country development status into account and are aimed at creating an enabling environment for healthcare integration.^1^ This further aligns with CAS theory, which views healthcare integration as emerging through self-organisation rather than central control.^43^ Thus, rather than enforcing integration, broad guiding principles to foster a positive environment for integration are recommended.^16,44^ According to a CAS approach, ‘top-down’ controls stifle creativity and negatively affect a health system’s ability to adapt.^16,44^ This approach is pertinent in the SA setting. The need for health system adaptation to the increasing prevalence of NCDs and the growing need for palliative care is apparent. Given the diverse contexts present within SA, a central control approach to EMS and palliative care would be futile, whereas applying the findings of this study as guiding principles would encourage integration cross-contextually.

## Limitations

This study contains limitations involving generalisability as a purposive sampling strategy was employed, resulting in a small group of participants largely from two provinces within SA. However, these participants work in diverse contexts within their provinces in both private and state sectors, including HE, management and patient-facing roles. While experts from other provinces were unable to take part because of scheduling constraints, the high proportion of participants from the Gauteng and Western Cape provinces are reflective of the greater EMS and palliative care resources in these areas.^37,38^ Study’s findings, including these contextual differences, have been discussed considering previous local and international research. Researcher bias may be present as the meeting facilitator knew many participants from the previous research and may have unintentionally influenced the group discussion through subconscious demonstration of personal opinions. However, researcher triangulation and member checking were performed to mitigate this. Finally, this study placed time constraints on participants and the findings of this study may not be comprehensive. Nevertheless, these findings align well with health system integration literature, represent the considerations of highly experienced SA providers, and therefore, remain applicable.

## Conclusion

Through use of a nominal group, consisting of experts from both EMS and palliative care, this study aimed to develop and prioritise approaches facilitating EMS and palliative care system integration within SA, thereby providing guidance for implementation in the country. We recommend the following highest ranked approaches as a practical starting point for EMS and palliative care integration in the country: (1) enable EMS to administer already prescribed medications, (2) EMS undergraduate training in palliative care, (3) improve EMS recognition of signs of dying at the end-of-life, (4) palliative care awareness for the EMS community, and (5) palliative care awareness for in-hospital healthcare providers, particularly those in emergency medicine. These approaches represent high impact, low-cost interventions, which are needed in the LMIC setting of SA. The implementation of these approaches will bring together currently siloed EMS and palliative care systems to enhance holistic care for those with palliative needs. Future research should aim at establishing the safety and efficacy of these interventions.
